# Distributed Dynamic Strain Sensing Based on Brillouin Scattering in Optical Fibers

**DOI:** 10.3390/s20195629

**Published:** 2020-10-01

**Authors:** Agnese Coscetta, Aldo Minardo, Luigi Zeni

**Affiliations:** Department of Engineering, University of Campania “Luigi Vanvitelli”, 81031 Via Roma 29, Aversa, Italy; aldo.minardo@unicampania.it (A.M.); luigi.zeni@unicampania.it (L.Z.)

**Keywords:** dynamic strain, distributed optical fiber sensors, stimulated Brillouin scattering

## Abstract

Over the past three decades, extensive research activity on Brillouin scattering-based distributed optical fiber sensors has led to the availability of commercial instruments capable of measuring the static temperature/strain distribution over kilometer distances and with high spatial resolution, with applications typically covering structural and environmental monitoring. At the same time, the interest in dynamic measurements has rapidly grown due to the relevant number of applications which could benefit from this technology, including structural analysis for defect identification, vibration detection, railway traffic monitoring, shock events detection, and so on. In this paper, we present an overview of the recent advances in Brillouin-based distributed optical fiber sensors for dynamic sensing. The aspects of the Brillouin scattering process relevant in distributed dynamic measurements are analyzed, and the different techniques are compared in terms of performance and hardware complexity.

## 1. Introduction

Recently, research and investment toward the development of distributed optical fiber sensors able to detect dynamic phenomena such as vibrations and sound waves have increased. The growing interest mainly comes from areas such as the oil and gas extraction companies [1], geophysics [2,3], and structural health monitoring [4]. In some applications, such as perimeter control and anti-intrusion, the simple detection of vibrations and their localization is sufficient, and this led to the development of the so-called distributed acoustic sensors (DAS) based on Rayleigh scattering [5,6]. On the other hand, when accurate and reliable measurements of the vibrations is required, sensors based on the Brillouin scattering can represent the right solution. This is especially true when structural analysis in aeronautic and civil applications is required to enable the identification of structural damages and pinpoint defects along large structures. In fact, the availability of an enormous number of spatially resolved data, typical of distributed optical fiber sensors, can make the difference. As an example, the detection of passive or actively induced vibrations in a structure can be exploited for non-destructive testing (i.e., online detection of cracks) without the need to dismantle the structure itself [7]. In addition, damage detection may get enormous advantage from distributed dynamic measurements, because changes in modal shapes may give precise indications on the position and severity of damage [8,9,10].

Conventional Brillouin scattering-based sensors suffer from a relatively long acquisition time (from seconds to minutes), basically due to two reasons: the first reason is that the intensity of the backward Brillouin scattering is weak, therefore many backscattered traces need to be averaged for adequate signal-to-noise ratio (SNR); the second reason is that the frequency offset between pump and probe beams must be swept, in order to obtain the Brillouin gain spectrum (BGS). Usually, several tens of Brillouin gain profiles must be acquired, to recover the temperature or strain distribution along the fiber [11]. In dynamic sensing applications, the acquisition time must be short enough to catch the relevant information. The required acquisition rate is very much dependent on the intended application, ranging from a few Hz in case of e.g., seismic monitoring [12] and wind turbine blade monitoring [13,14], up to several kHz in case of e.g., railway traffic monitoring [15] and pipeline leakage monitoring [16].

To reach an acquisition rate adequate for dynamic sensing applications, several techniques addressing the second aspect have been developed, i.e., they are aimed to eliminate (or at least attenuate) the requirement of scanning the probe frequency. On the other hand, it is clear that any optimization of the experimental configuration that increases the SNR (e.g., based on the use of a narrow linewidth laser [17], optimized detection schemes [18,19], or more sophisticated methods for the suppression of the polarization noise [20]) is also beneficial to dynamic measurements, as it permits the cutting down of the averaging factor. For example, the simple (but costly) adoption of a polarization-maintaining (PM) fiber as the sensing fiber, leads to obvious advantages in terms of acquisition speed, as it eliminates the averaging requirements usually associated with the polarization scrambling of the pump, and required to compensate the Brillouin gain fluctuations due to the fiber birefringence. In this review, we focus our attention on the schemes aimed at reducing the acquisition time associated with the probe frequency sweep, either by reducing the number of swept frequencies, or by accelerating the sweep process. Nonetheless, it is understood that any strategy that leads to an SNR improvement (with consequent reduction or even avoidance of averaging) can be adopted to raise up the acquisition rate, making it compliant to he intended application. 

The paper is organized as follows: after a brief recall of the Brillouin analysis and the discussion on the main factors limiting its application to dynamic sensing, the main technical solutions that have been devised to overcome the problem will be described along, in some cases, with illustrative experimental achievements. 

## 2. Brillouin Analysis for Distributed Sensing

**Figure 1:**
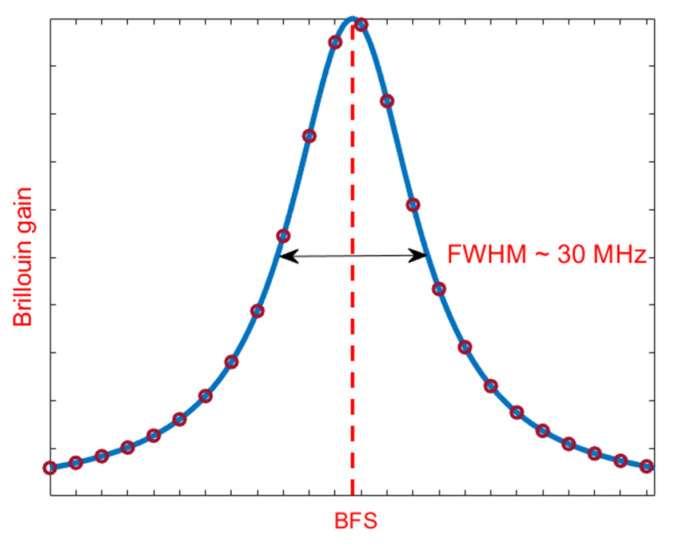
Brillouin Gain Spectrum in a conventional silica fiber. The red circles represent the frequencies acquired for BFS extraction.

When a pump light in injected into an optical fiber, it interacts with the quantized vibrational modes of molecules in the matter (the so-called acoustic phonons). They can be regarded as acoustic waves, inducing periodic variations in the refractive index of the fiber due to the elasto-optic effect. These traveling refractive index variations act as a diffraction grating, scattering the incident light in all directions. In the backward direction, the frequency of the scattered light is downshifted from that of the incident light, by a quantity equal to the frequency of the acoustic wave (the so-called Brillouin Frequency Shift, BFS). When a probe light, frequency shifted from the pump light by the BFS, is injected from the opposite side of the fiber, an acoustic wave much more intense than those thermally excited is generated by electrostriction, leading to a stronger backscattered signal. The underlying phenomenon is known as stimulated Brillouin scattering (SBS), and has been employed to realize Brillouin lasers [21], amplifiers [22], slow-light systems [23,24], as well as for the implementation of distributed sensors able to sense temperature and strain changes over large distances [25,26]. The dependence of the backscatter amplitude from the frequency shift between pump and probe waves follows a typical Lorentz function (see Figure 1), centered at the BFS. The latter is given by:

(1) $$BFS=\frac{2n_{eff}V_{A}}{\lambda }$$

where *V_A_* is the velocity of the acoustic waves, *n_eff_* is the effective refractive index, and *λ* is the optical wavelength. Both *V_A_* and *n_eff_* depend on the temperature and strain of the fiber, therefore the changes in these two parameters can be measured by monitoring the changes in the BFS. The BFS is 10 ÷ 11 GHz in silica fibers at λ = 1550 nm, while temperature and strain sensitivities are ∼ 1 MHz/°C and ∼50 kHz/µε, respectively. 

To get information about the distribution of the BFS along the fiber, some form of modulation of the pump light is required. A comprehensive review of the most relevant demodulation techniques for Brillouin sensing can be found in Ref. [27]. Here, we briefly recall the three main approaches: the Brillouin Optical Time-Domain Analysis (BOTDA), the Brillouin Optical Frequency-Domain Analysis (BOFDA), and the Brillouin Optical Correlation-Domain Analysis (BOCDA). 

The BOTDA makes use of a pulsed pump, therefore it strictly resembles the conventional optical time domain reflectometry. The spatial resolution, i.e., the capability to discriminate between the BFS in adjacent positions, is determined by the temporal width of the pulse injected into the fiber. The detected BGS is the convolution between the natural BGS (with a linewidth of about 30 MHz) and the pulse spectrum. Therefore, pulse durations less than about 10 ns led to a broadened BGS and, consequently, a less accurate BFS estimate. When a submeter spatial resolution is required, some form of pre-activation of the acoustic wave is usually adopted to measure the BFS with adequate accuracy [28,29,30,31]. As regards the acquisition time, each Brillouin gain profile (i.e., each point in the BGS frequency domain) requires an (ideal) acquisition time equal to the roundtrip time of the pulse over the fiber length, i.e., $T=\frac{2n_{g}}{c}L$, where *L* is the fiber length and *n_g_* is the group velocity of the pulse. For example, the roundtrip time for a 100-m fiber length is ∼1 µs, unveiling a maximum acquisition rate of 1 MHz if no averaging nor probe frequency sweep is performed. 

**Figure 2:**
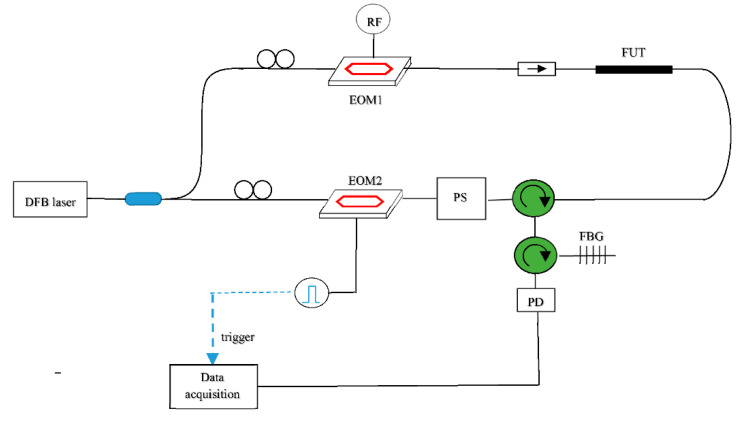
Conventional BOTDA configuration. PS: polarization scrambler; FBG: fiber Bragg grating. EOM: Electro-optic modulator. PD: photodetector.

The basic setup for BOTDA measurements is shown in Figure 2. A single laser source (typically, a distributed feedback diode laser), is employed for pump and probe beam generation. The frequency-shifted probe is generated through an electro-optic modulator (EOM1) biased near zero transmission point, driven by a radiofrequency signal to realize a dual sideband modulation. The pump is pulsed using a second electro-optic modulator (EOM2), also biased near zero transmission point and driven by an electrical pulse. The fiber Bragg grating in the receiver path acts as a reflection bandpass optical fiber, selecting one of the two sidebands generated by EOM1 (the one at lower frequency, typically). Finally, a polarization scrambler (PS) is inserted into the pump path in order to remove (by averaging) the signal fluctuations associated with the dependance of the Brillouin gain from the lightwaves polarization. The pulse generator acts as a trigger for data acquisition, which is generally carried out in average mode to reach an adequate SNR. The conventional BOTDA measurement process requires the pump/probe frequency shift to be swept around the BFS of the fiber, to reconstruct the BGS shown in Figure 1. This scan is performed step by step: the frequency shift (i.e., the radiofrequency applied to EOM1) is set, and then a certain number of Brillouin gain profiles are acquired, cumulated (averaged) and stored into memory. Then, a new frequency shift is set, and a new collection of Brillouin gain profile acquisitions is done. The process terminates when an adequate number of frequency shifts (i.e., data points in the BGS frequency domain) have been acquired. 

A different method, working in the frequency domain (the so-called BOFDA), makes use of an intensity- or phase-modulated pump wave [32,33,34]. Sweeping the modulation frequency over a proper range and a proper step (which depend on the measurement range and intended spatial resolution), the modulation impressed on the emerging probe wave is captured by a vector network analyzer (VNA). In this case, the data acquired by the VNA must be inverse Fourier transformed, to retrieve the Brillouin gain profile along the fiber. Cm- or even mm-scale spatial resolutions are easily achieved in BOFDA sensors, making them especially attractive in the aerospace industry [35,36]. The acquisition of each Brillouin gain profile takes a time equal to the inverse of the resolution bandwidth (RBW) of the VNA. The RBW is the bandwidth of the bandpass filter used for data acquisition and is related to the fiber length by the Nyquist’s theorem. Therefore, the BOFDA method has theoretically the same performance as the BOTDA method in terms of acquisition time. However, the use of a small-signal modulation for the pump implies that an adequate SNR is usually achieved only by setting an RBW (at least) one order of magnitude narrower than that dictated by Nyquist, making BOFDA sensors less attractive than BOTDA sensors for dynamic sensing applications. 

Besides time-domain and frequency-domain detection schemes, a correlation-based technique based on frequency-modulated continuous-wave pump and probe lights has been demonstrated as well [37]. The Brillouin Optical Correlation Analysis (BOCDA) provides excellent spatial resolution capabilities (down to the mm scale), at the expense of the maximum number of sensing points, which is typically limited to a few hundreds. For example, a 1-cm spatial resolution implies a maximum sensing length of a few meters. Thus, the correlation method is suitable in the monitoring of relatively small-sized structures, such as composite material parts in the automotive, nautical, and aerospace industries [38]. As regards the acquisition speed, the following argument can be followed: the measurement range *d_m_* in BOCDA sensors is given by $d_{m}=\frac{v_{g}}{2f_{m}}$ [39], where *v_g_* is the group velocity, and *f_m_* is the sine-wave modulation applied to the pump and probe waves for the synthesis of a correlation peak. Assuming that the Brillouin gain (i.e., the probe amplification) is captured by use of a lock-in amplifier with a time constant 50 times larger than the inverse of *f_m_* [40], the maximum acquisition rate *f_s_* will be given by to $\frac{v_{g}}{100\times d_{m}}$. For example, for a fiber length of 100 m, the maximum acquisition rate is 20 kHz. However, it must be kept in mind that this rate only refers to the measurement over a single position of the fiber under test (FUT). Measurement over more locations leads to a proportionally longer acquisition time. 

Independently of the chosen detection scheme (time domain, frequency domain, or correlation domain), the need to scan the pump–probe frequency shift over ~200 frequencies is often the bottleneck of the acquisition rate. Consequently, methods to overcome this limitation have been devised and are described in the following sections. 

## 3. Dynamic Sensing Based on BOCDA

**Figure 3:**
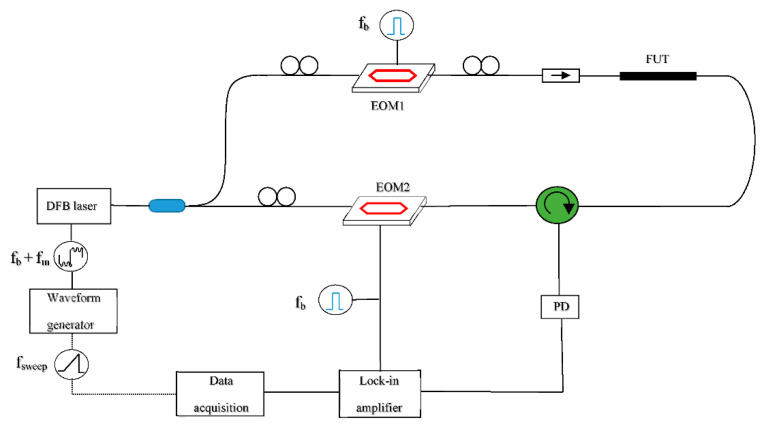
Experimental setup employed for BOCDA measurements; EOM, electro-optic modulator; PD, photodiode; FUT, fiber under test (Adapted from Ref. [39]).

Interestingly, the first report of a relatively high acquisition rate was demonstrated with a BOCDA configuration [39], which, according to what discussed earlier, is the less favorable for distributed dynamic sensing (due to the necessity to scan the correlation peak over each sensed position). As a matter of fact, the method was applied to detect the dynamic strain over a single location. The configuration is shown in Figure 3. The scheme implements a modified BOCDA configuration, in which the frequency-shifted probe wave is generated by switching the laser frequency, rather than applying an external modulation to the laser. In any case, this modification was only intended to simplify the experimental configuration, having no effect on the maximum permissible acquisition rate. The laser current waveform was formed by superimposing a sine-wave modulation at a few MHz, required to localize the SBS interaction in a specific section of the FUT, to a 100-kHz square wave required to alternate the pump and probe frequencies. By this method, the authors reported the acquisition of strain over a single position along a 20-m long FUT, at a maximum sampling rate of 1 kHz and spatial resolution of 10 cm. 

**Figure 4:**
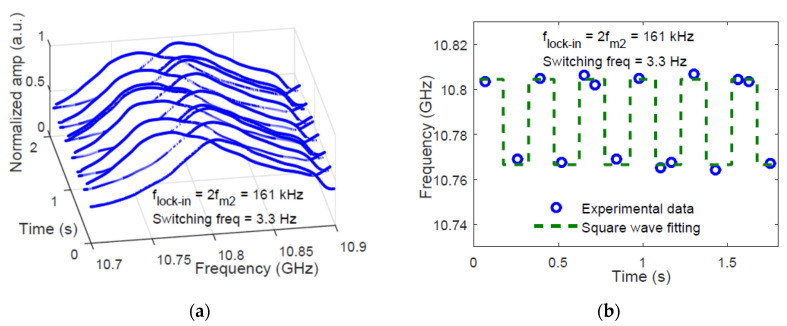
BOCDA measurements at two sensing locations simultaneously. (**a**) BGS acquired in the location subjected to BFS changes. (**b**) Corresponding BFS changes [42].

As discussed earlier, whenever dynamic sensing over more locations is required, BOCDA systems are not the best solution, as only one position can be sensed at a time. In other words, the sampling rate is scaled down proportionally to the number of sensed locations. More advanced schemes have been demonstrated, in which the amplitude of the backscatter signal is measured in multiple locations simultaneously. These schemes include the use of a pulsed pump, to discriminate between multiple correlation peaks along the FUT [41], or multi-tone modulation of the laser light [42]. In the latter case, an electrical spectrum analyzer (ESA), locked at twice the modulation frequency, detects the Brillouin gain changes in the position corresponding to that modulation frequency. In Ref. [42], the measurement of the dynamic strain at two locations simultaneously, was demonstrated in a 1100 m long fiber at a spatial resolution of 60 cm. The time required to perform the BGS acquisition in both positions was 210 ms (in case of 3-MHz sampling step for the BGS), or 65 ms (in case of 10-MHz sampling step) (see Figure 4). Still, the acquisition rate was rather low (a few Hz), compared to the inverse of the roundtrip time for the given fiber length, suggesting a not optimized measurement SNR of the experimental scheme. Also, the method requires an independent detection channel for each sensing location. In other words, two independent ESAs (or two independent channels of the same ESA) are required for two correlation peaks (i.e., two sensed locations), making the method hardly scalable.

A different method, referred to as differential frequency modulation BOCDA, has been also proposed to fast scan the correlation peak (i.e., the sensed position) over the entire fiber [43]. The setup is very similar to the one shown in Figure 3, with the difference that the modulation frequency applied during pump generation is slightly detuned (a few kHz) from the modulation frequency applied during probe generation. In this way, the correlation peak moves continuously along the fiber at a constant speed. By applying this method, the authors have demonstrated the measurement of the BFS along the entire fiber length (100 m), at a spatial resolution of 80 cm and an acquisition rate of 20 Hz. Although the method is effective in moving the sensed position over the entire fiber, the acquisition rate is severely impaired, compared to the conventional BOCDA method with a single sensing position. 

A successive improvement of the BOCDA method, still aimed at increasing the acquisition rate, has been presented in [44]. Here, the authors made use of a voltage-controlled oscillator (VCO) in order to change rapidly the frequency of the probe, reaching in this way an acquisition rate as high as 5 kHz over a fiber of 6 m and at a spatial resolution of 3 cm. However, the measurement was still limited to maximum five (arbitrarily selected) sensing locations simultaneously.

## 4. Slope-Assisted BOTDA (SA-BOTDA) Methods

**Figure 5:**
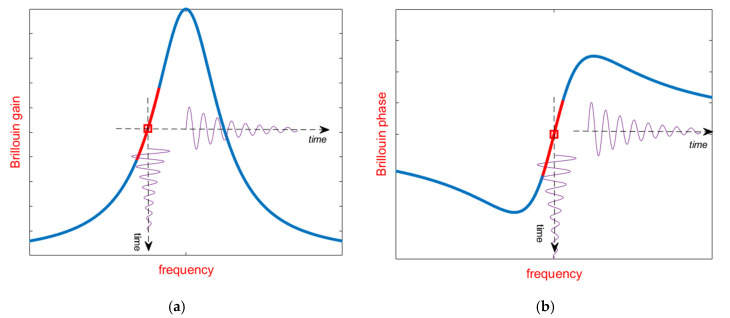
Principle of operation of the SA-BOTDA method: the strain-induced spectral shifts of the BGS (**a**) or the BPS (**b**) can be regarded as temporal variations of the probe frequency around its working point.

Although the methods discussed in the previous sections still rely on the measurement of the Brillouin gain at various pump–probe frequency shifts, slope-assisted (SA) techniques are based on the measurement of the Brillouin gain for a fixed pump–probe frequency shift, chosen in order to lie within the rising or the falling slope of the BGS (see Figure 5a). In such a way, the BFS temporal changes are directly transferred to the amplitude of the backscattered trace, at least for BFS variations small enough with respect to the BGS linewidth. This method somewhat resembles the demodulation technique used in fiber Bragg grating (FBG)-based dynamic sensing, in which the laser wavelength is locked on the mid-reflection wavelength of the FBG reflection spectrum [45]. Of course, the concept of SA measurements can be equally applied to the Brillouin Phase Spectrum (BPS) [46,47,48] (see Figure 5b), or to the gain-to-phase ratio [48,49]. Lifting the measurement process from the need to scan the pump–probe frequency, provides a simple and effective way to speed-up the acquisition rate (at the expense of the dynamic range), which will be only limited by the fiber length and the averaging factor [50]. Furthermore, the SA-BOTDA method can be paired with any acoustic pre-activation method (such as the pulse-pair differential method), in order to push the spatial resolution below to the 1-m limit set by the phonon lifetime [51].

**Figure 6:**
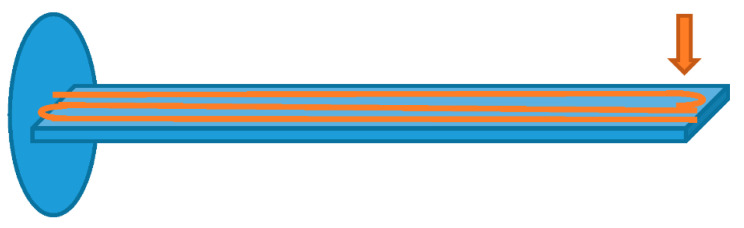
Cantilever beam used for modal analysis experiments [52].

In the first demonstration of this idea, the dynamic strain was acquired at a sampling rate of 200 Hz over a fiber length of 30 m, at a spatial resolution of 3 m and with a dynamic resolution of 27 $\mu \varepsilon /\sqrt{Hz}$ [50]. Although in that case a pulsed probe was let to interact with a counter-propagated pulsed pump, restricting the measurement at a single location at a time, the method can be equally applied using a conventional cw probe, allowing simultaneous measurements over the whole fiber. As an example of truly distributed and dynamic strain measurements, a modal analysis of an aluminum beam excited via a magnetic shaker was reported in Ref. [52] (see Figure 6). The setup used for the experiments is the conventional BOTDA shown in Figure 2. The SA-BOTDA measurement was carried out by fixing the probe frequency shift (i.e., the RF frequency applied to the EOM) in order to lie within the rising (or falling) slope of the local BGS. 

**Figure 7:**
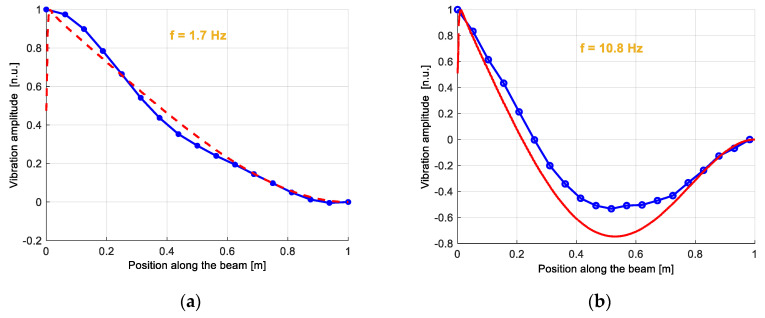
Dynamic strain amplitude related to the (**a**) first and (**b**) second mode of the cantilever beam. Mode frequencies are 1.7 Hz are 10.8 Hz, respectively. The blue lines represent the mode shapes measured using the SA-BOTDA method, while the red lines represent the FEM result [52].

The cantilever beam had a length of 1 m, a width of 3 cm and a thickness of 1cm. The fiber was glued along three parallel directions of the beam, to capture the strains along multiple lines. The dynamic strain magnitude at 1.7 Hz and 10.8 Hz (corresponding to the first two modes of the structure), measured along the middle fiber, are shown in Figure 7, together with the results of a finite element method (FEM) analysis. Discrepancies between experimental and numerical results are mostly attributed to the limited (50 cm) spatial resolution of the measurements. Please note that each experimental point in the mode shapes of Figure 7 was calculated as the magnitude of the fast Fourier transform (FFT) of the dynamic strain signal acquired in that position, over a time period of 20 s. Of course, the acquisition of the strain temporal changes over a temporal window of 20 s, under constant vibrating conditions, greatly enhances the SNR compared to the capture of a single-shot, transient phenomenon. 

The SA-BOTDA method was further employed by the same authors for the modal analysis [9] and defect identification [10] of a composite plate, and for railway traffic monitoring [53]. In the latter case, the method was employed to measure the strain induced by train passage along a rail track, over a distance of 60 m and at an acquisition rate of 31 Hz. This was sufficient to detect the train passage and extract several information from the acquired data, such as train speed, number of axles, interaxle distances, and axle load. 

Despite its advantages, the SA-BOTDA method has its own limitations: first, the method requires the probe frequency to lie within the slope of the BGS (or BPS as well). In general, this condition cannot be met in all fiber positions, because each position may exhibit a different BFS due to strain/temperature spatial nonuniformities. Another limitation is that even when the probe frequency is exactly tuned to the middle of the BGS linear slope, the maximum strain variation is limited to about a few hundred µε for faithful frequency/amplitude conversion (see Figure 5). Third, the system is more susceptible to noise compared to the conventional BOTDA method, in which the BFS is recovered by processing (e.g., by quadratic fitting) the entire BGS. For example, operating with a typical laser linewidth of 1 MHz, the SA method directly translates the laser phase noise in a rms strain error of about 20 µε [54]. Finally, the SA-BOTDA method recovers the strain from the amplitude (rather than the frequency) of the backscattered signal, making it prone to errors related to laser power variations.

**Figure 8:**
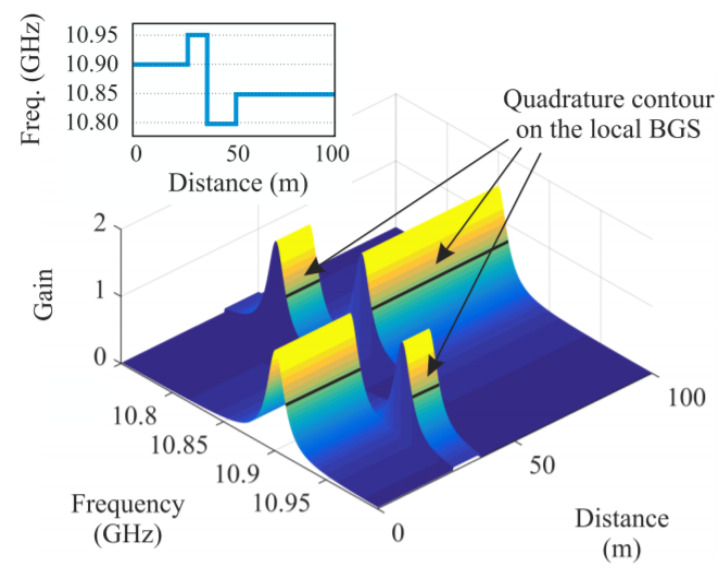
Fast changing the probe frequency allows the latter to lie within the slope of each local BGS, even in case of spatial nonuniformities [55].

**Figure 9:**
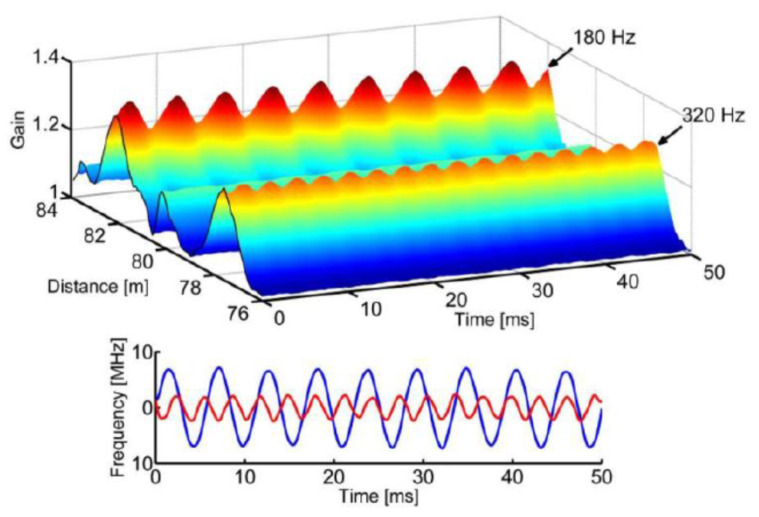
(**Top**) Brillouin gain variations acquired along an 85-m long fiber with two sections subjected to dynamic strain. (**Bottom**) Corresponding BFS changes in the two positions [55].

As regards the first limitation, a modification of the SA technique has been demonstrated in Ref. [55], relying on the use of a specially synthesized probe wave, whose frequency is rapidly changed by use of an arbitrary waveform generator (AWG) in order to lie along the slope of the BGS in each fiber position (see Figure 8). Exploiting this method, the authors have performed dynamic strain measurements over an 85 m long fiber at a spatial resolution of 1.5 m. The dynamic strain detected in two positions, vibrating at 180 Hz and 320 Hz, respectively, and with a static BFS differing by 120 MHz (thus well above the constraint imposed by the conventional SA-BOTDA method), are shown in Figure 9 (data were 1-kHz lowpass filtered for SNR enhancement).

As regards the dynamic range issue, a simple solution stems from the use of pump pulses shorter than the phonon lifetime (~10 ns). In fact, this leads to a broadening of the BGS linewidth (which scales with the inverse of the pulse duration), which enlarges the dynamic range while also helping in equalizing the dynamic sensitivity along the fiber [56]. This also has a positive effect on the spatial resolution. On the other hand, shortening the pulse width reduces the backscattered signal amplitude and reduces the BGS slope as well. These two factors together may eventually compromise the measurement SNR. More sophisticated solutions have been demonstrated, in which the pump pulse spectrum is properly engineered for dynamic range extension under the same spatial resolution [57,58]. 

**Figure 10:**
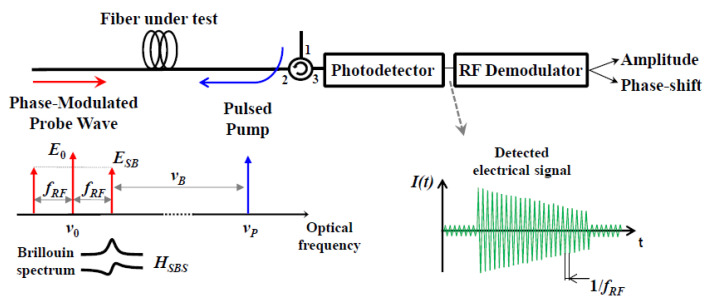
SBS interaction between a pulsed pump and a phase-modulated probe wave for dynamic strain measurements [47].

Finally, as regards the sensitivity to pump power fluctuations, measurement configurations independent of the pump power have been demonstrated as well. In Ref. [47], a configuration capable of extracting the dynamic strain from the BPS, rather than the BGS, was demonstrated. The conceptual scheme is shown in Figure 10.

**Figure 11:**
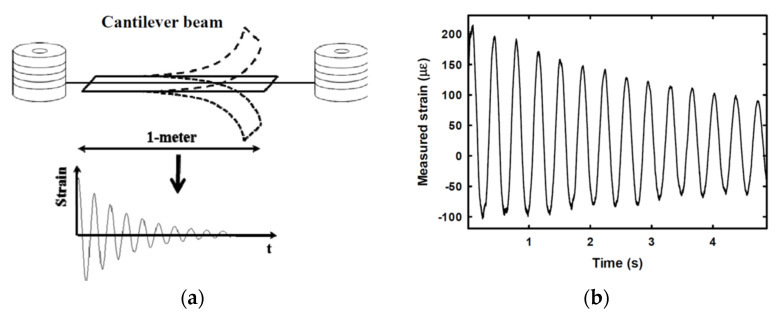
Experimental demonstration of dynamic strain measurement (**b**) over a 1-m cantilever beam subjected to vibration (**a**), carried out using the setup of Figure 10 [47].

The method makes use of a phase-modulated probe field, whose modulation frequency is much higher than the BGS bandwidth. In this way, only one modulation sideband falls within the BGS (and BPS) generated by the pump. The gain and phase-shift induced by SBS on this sideband modulates the heterodyne signal produced on a photodetector placed at the fiber output, from which the phase modulation induced by SBS is easily extracted. The use of the BPS, rather than its amplitude counterpart, provides a measurement of the BFS shift (i.e., strain) not affected by pump power variations. The use of the BPS for Brillouin measurements is also beneficial for long-range measurements, as it provides BFS measurements immune to the nonlocal effects deriving from the frequency-dependent depletion of the pump pulse [59]. The strain acquired at a sampling rate of 1.66 kHz along a piece of fiber attached to a 1-m long cantilever beam subject to vibrations is shown in Figure 11 (the total fiber length was 160 m in this experiment).

**Figure 12:**
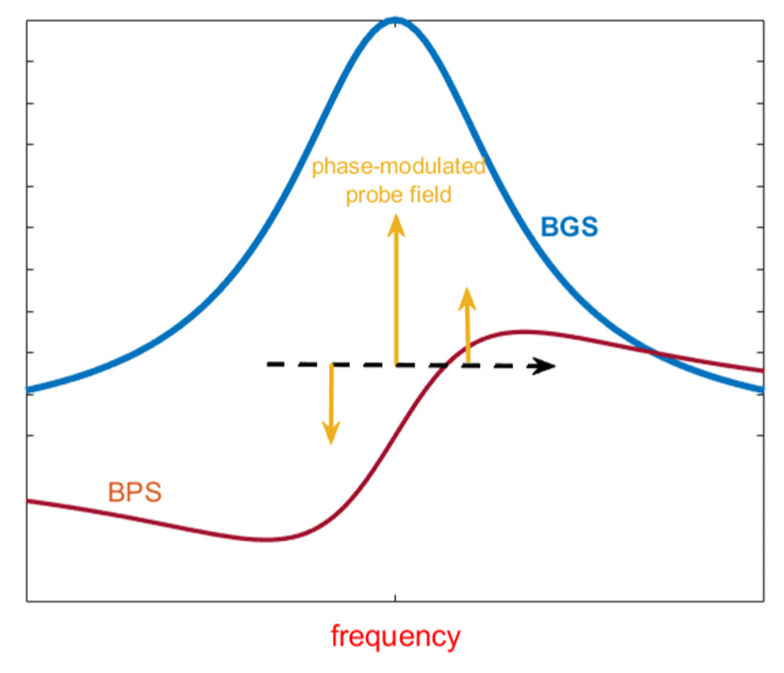
Schematic representation of the interactions involved in the heterodyne slope-assisted method [60]. The BGS and BPS affect both the carrier and the two first-order sidebands of the phase-modulated probe, leading to the appearance of an RF beat note on the transmitted probe intensity.

A somewhat similar approach has been reported in Ref. [60], where the phase of the probe beam was modulated at a much lower frequency. In this case, both carrier and first-order sidebands fall within the BGS and then interact with the pump light (see Figure 12). Tuning the carrier frequency at the BGS resonance and setting a modulation frequency equal to half the BGS linewidth, the two sidebands experience the same gain but opposite phase-shift. Thus, a partial phase-to-amplitude conversion appears on the emerging probe intensity. In other words, the probe intensity contains a self-heterodyne beat note at the same frequency of the phase modulation.

**Figure 13:**
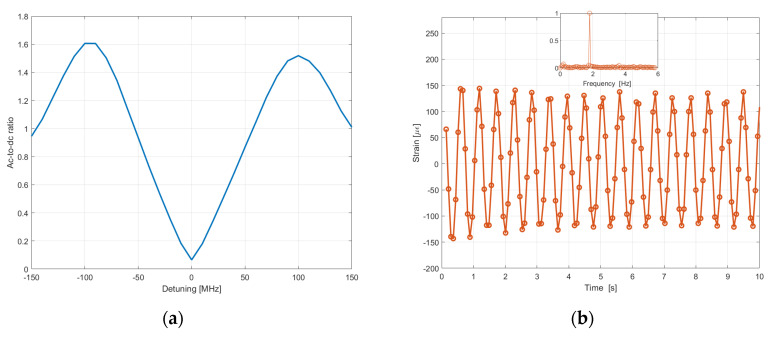
(**a**) Amplitude of the heterodyne signal normalized to its dc value, computed for a pulse duration of 10 ns, as a function of detuning from resonance (**b**) Dynamic strain measured on a 1-m long cantilever beam subjected to vibration. (The inset shows the FFT of the acquired waveform [60]).

When an external perturbation (strain) modulates the BFS, the amplitude of this tone grows linearly with the applied strain. More importantly, the ratio between the amplitude of this beat note, and the DC component of the transmitted probe wave is independent of the pump power. We show in Figure 13a the ac-to-dc ratio, calculated as a function of the frequency detuning from resonance, and for a pump pulse duration of 10 ns. The plot reveals that tuning the carrier frequency about half a BGS bandwidth away from the resonance, a linear slope is available for dynamic strain measurements. 

Figure 13b shows the heterodyne signal converted in strain units, acquired by attaching the fiber on a cantilever beam vibrating at the frequency of 1.8 Hz, and with an acquisition rate of 13 Hz. Compared to the conventional SA-BOTDA method, the heterodyne SA-BOTDA method provides a better SNR thanks to the fact that the signal is shifted to a higher frequency (therefore reducing the influence of the low frequency laser noise). Furthermore, compared to Ref. [47] the technique has the advantage of not requiring the use of a high-bandwidth (~ GHz) detector for the extraction of the phase shift. 

**Figure 14:**
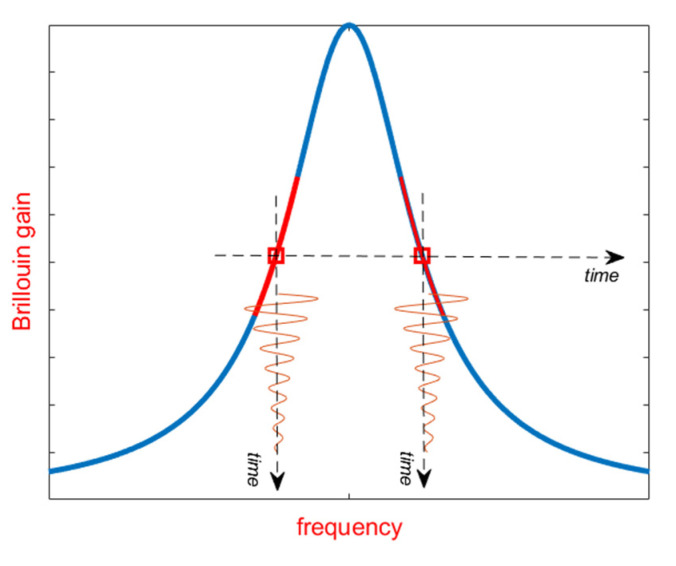
In the dual slope-assisted BOTDA method, the probe frequency is switched between the two slopes, while the dynamic strain is extracted from the ratio between the signals acquired from the two slopes [61].

In Ref. [61] a dual SA method has been demonstrated for pump power independent dynamic strain measurements. In this method, the carrier frequency of the probe wave is switched between the two slopes of the BGS, while the useful signal is calculated as the ratio of the measurements taken on the two slopes (see Figure 14). Ideally, when no perturbation is applied to the fiber, this ratio is unitary: when a strain is applied, the signals from the two slopes change in opposite way, thus their ratio changes as well. Instead, when the pump power changes, both signals scale by the same factor, therefore their ratio remains unchanged. Although the dual SA method provides pump power independence, it does not impact the dynamic range, which is still limited to a few hundred µε. Furthermore, the necessity to switch from one slope to the other reduces the maximum acquisition rate by half.

**Figure 15:**
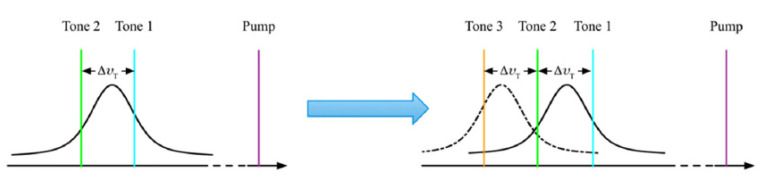
Extension of the measurement range by multi-slope-assisted BOTDA [62].

An extension of this technique has been demonstrated by Dexin et al. [62], in which the probe frequency is stepped consecutively by a quantity equal to the BGS linewidth (see Figure 15). This method extends the dynamic range, as the BFS is no more bounded to lie within the slope of a single BGS. As with the dual slope method, the drawback of the multiple slope BOTDA method is a reduction of the maximum acquisition rate by a factor equal to the number of slopes employed for the measurement. 

## 5. Frequency-Sweep Free Methods

**Figure 16:**
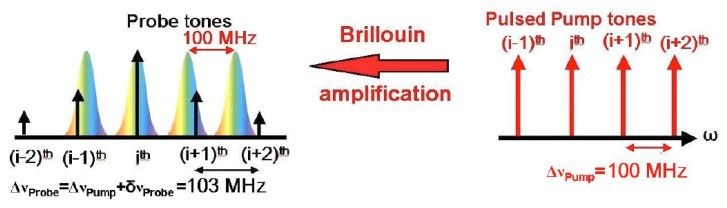
SBS interaction between a multi-tone pump wave and a multi-tone probe wave as done in the frequency-sweep free method [63].

The methods discussed in the previous paragraph remove the need to scan the pump–probe frequency shift, by probing the Brillouin gain/phase at only one (or a few ones) frequencies. A different approach relies on the use of a multi-tone pump (and/or probe) wave, in such a way that the Brillouin gain is recovered at different frequencies simultaneously. The first demonstration of this technique, referred to as frequency-sweep free method, has been reported by Voskoboinik et al. [63]. The basic concept is illustrated in Figure 16. Instead of using a single-frequency pulsed pump, the method relies on the use of a discrete number *N* of pump pulses, equally spaced in the frequency domain by a quantity significantly larger than the BGS linewidth (100 MHz, typically). Each pump tone generates a spectrally shifted BGS. A number *N* of probe tones are simultaneously launched into the sensing fiber from the opposite side. These tones are downshifted from the pump tones by approximately the BFS but are spectrally shifted by a slightly larger quantity. As each probe tone lies in a different position of its corresponding BGS, the acquisition and spectral analysis of the probe signal intensity at the exit of the FUT permits recovery of the BGS in multiple frequencies simultaneously. 

**Figure 17:**
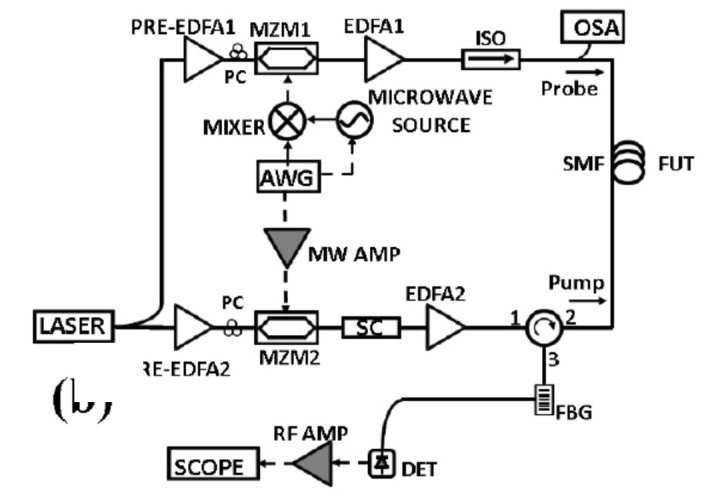
Experimental setup employed for sweep-free BOTDA measurements [63].

The experimental setup used in Ref. [63] is reported in Figure 17. The configuration is very similar to the conventional BOTDA scheme, with the only exception that both pump and probe beams are now intensity-modulated to realize the multi-tone scheme.

**Figure 18:**
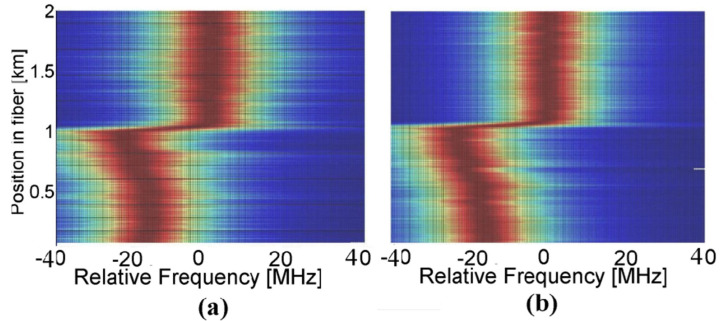
Measurement of the BGS along a 2-km FUT using (**a**) the frequency-sweep method, with 30 frequency tones spanning 90 MHz, reconstructed at a spatial resolution of 50 m; or (**b**) Using a classical BOTDA with 3MHz sweeping step [63].

Figure 18 shows the BGS profiles acquired along a 2-km FUT, composed by two fiber spools spliced at 1000-m. The figure compares the result of the sweep-free method (Figure 18a), implemented with a 30-tones frequency comb and a frequency shift between each pump–probe pair differing by 3 MHz from the next one, with the result of the conventional BOTDA (Figure 18b) using the same granularity (3 MHz). The results are perfectly consistent, which means that the sweep-free method, in which a certain number of pump–probe pairs is let to interact during each step, provides similar performance as the conventional BOTDA, where a single probe/probe pair interacts at each step, with a speed-up factor equal to the number of pump–probe pairs (30, in this case), because each pair replaces one sweeping step required in the classical BOTDA.

In practical applications, the main drawbacks of the sweep-free method are a higher complexity of the measurement setup, due to the necessity to modulate both pump and probe beams with multifrequency waveforms, but especially the fact that it adds a trade-off between the granularity in the BGS reconstruction and the spatial resolution. In fact, the method requires that the probe signal is analyzed over time windows at least equal to the inverse of the BGS sampling step: this limits the spatial resolution to a few tens of meters for adequate BGS granularity, independently of the duration of the pump pulses. Please note that a similar limitation afflicts the techniques described in Refs. [64,65], where a single-tone pump pulse and a multi-tone probe are employed. For example, in Ref. [65] a frequency comb composed by about 1000 tones separated by 1.95 MHz, was employed as the probe beam. Due to interaction with a counter-propagating, single-tone pump pulse, each tone in the probe wave experiences a different Brillouin gain, thus the frequency comb is reshaped at the exit of the fiber. Analyzing the transmitted probe over successive time slots, the BGS related to each corresponding fiber portion can be reconstructed. As the duration of each time slot is inversely proportional to the BGS frequency granularity, a typical spatial resolution of tens of meters results for adequately fine spectral resolution. As an example, in Ref. [65] the actual spatial resolution was limited to about 50 m due to the duration (500 ns) of the time slots employed for demodulating the received frequency combs. In Ref. [66], the same authors have proposed a variant of this method, in which the transmitted multi-tone probe wave is coherently received, i.e., it is mixed with a local oscillator. Compared to the method presented in Ref. [65] based on direct detection, the use of coherent detection permits the retrieval of the BPS, which has a linear shape near the BFS (see Figure 5b). Furthermore, the boost in the detected signal provided by coherent detection allowed the authors to perform the measurements without averaging, therefore reaching an ultimate acquisition rate only limited by the fiber length (e.g., 10 kHz for a 10-km long fiber spool). Still the main drawback is the limited spatial resolution (about 50 m also in this case).

## 6. Fast Frequency Sweeping Methods

**Figure 19:**
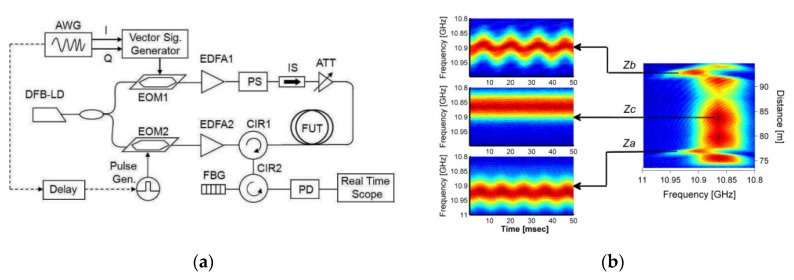
(**a**) Experimental setup for fast frequency sweeping BOTDA; (**b**) BGS acquired in one fixed and two vibrating locations of the FUT [67].

Fast frequency sweeping methods provide better acquisition rates compared to the conventional BOTDA method, as they compress (or eliminate) the settling time required to switch the probe frequency during the measurement. In fact, as discussed in Section 2, the measurement process in the BOTDA method requires the Brillouin gain profile to be acquired for each preset pump/probe frequency shift. The time required to switch the probe frequency between two consecutive measurements may be in the order of milliseconds, and must be counted as many times as the number of scanned probe frequencies. Thus, it can significantly deteriorate the maximum acquisition rate. The first demonstration of this method is by Peled et al. [67]. The authors employed an AWG to fast switch the probe frequency among 100 scanning frequencies. The experimental setup is shown in Figure 19a. Compared to the conventional BOTDA method, the probe frequency is fast swept through a vector-modulated microwave source, with the in-phase and quadrature modulation signals provided by a two-channel AWG pre-loaded with the frequencies chosen to obtain the BGS. An acquisition rate of 10 kHz was demonstrated over a fiber length of 100 m and at a spatial resolution of 1 m (see Figure 19b).

**Figure 20:**
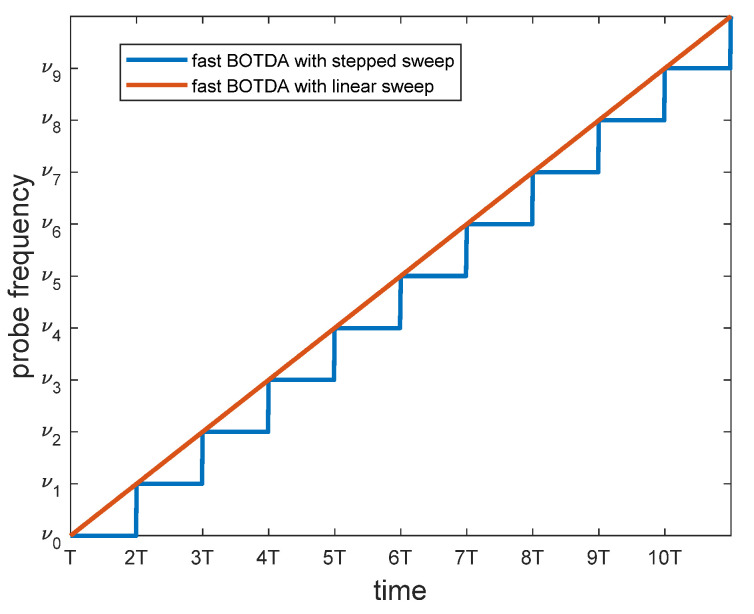
Probe frequency sweep used for stepped sweep [67] and linear sweep [69] BOTDA measurements. *T* represents the time slot allocated for the acquisition of each Brillouin gain profile (in the ideal case, e.g., no averaging, *T* corresponds to the roundtrip time of the pump pulse over the fiber length).

Although effective in cutting down the acquisition time, this method has the disadvantage of requiring a fast AWG to scan the probe frequency. The AWG has less stringent requirements when a second-order modulation scheme is adopted for the frequency-shifting device, i.e., by biasing the EOM at maximum transmission and carefully choosing the RF amplitude [68]. An alternative (and cost-effective) approach consists of sweeping the probe frequency in a linear, rather than step-like, fashion [69], through the use of a frequency-agile RF generator. In this case, the probe frequency is linearly swept, so that at the end of the linear sweep the whole BGS is obtained. The scan rate is so slow that the pump pulse “sees” an almost constant probe frequency during its propagation, so that the emerging probe intensity can be uniquely associated with a single point in BGS frequency domain. Compared to the original fast frequency-sweep method proposed in Ref. [67], this method is simpler as it requires a frequency-agile microwave source, instead of an AWG and a microwave source with vector modulation capabilities. Figure 20 compares the frequency sweep applied to the probe in the fast BOTDA method proposed in Ref. [67], with one employed in Ref. [69].

**Figure 21:**
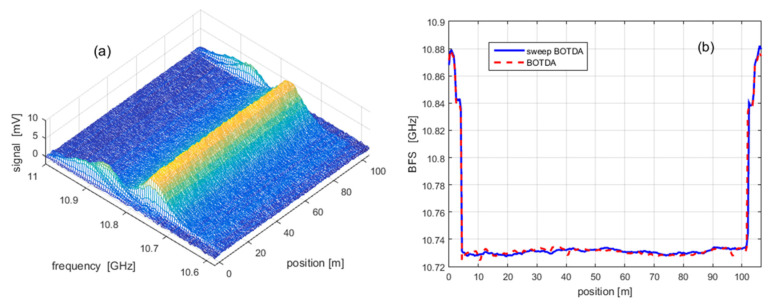
**(a)** BGS acquired by using a linearly swept probe frequency, in a sweep time of 33 ms; (**b**) Comparison of the BFS profile acquired using either the FAST BOTDA method with a linearly swept frequency, or the conventional BOTDA method.

Figure 21a reports the BGS distribution acquired using a linearly swept probe frequency, with a sweep time of 33 ms, a fiber length of 100 m and a spatial resolution of 1 m. In Figure 21b, the quality of the BFS reconstruction is validated against the BFS profile acquired using the conventional BOTDA, with the significant difference that the latter is acquired in a time of about 0.5 s (more than ten times longer), mainly due to the settling time of the microwave source. 

**Figure 22:**
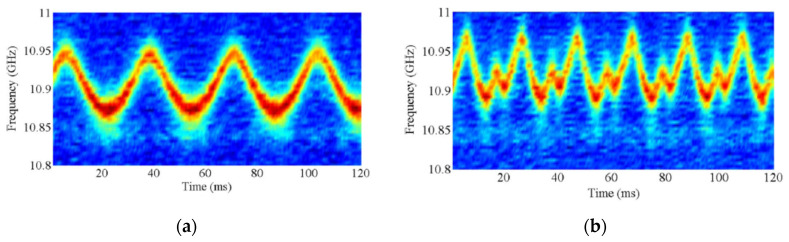
BGS acquired using the fast BOTDA method combined with the differential pulse-pair technique [68]. The vibrating fiber was excited at a frequency of (**a**) 33.3 Hz or (**b**) 50 Hz.

Fast BOTDA methods can be also combined with the differential pulse-pair method originally proposed in Ref. [29], to enhance the spatial resolution down to the cm-range. In brief, the differential pulse-pair method involves the measurement of the Brillouin gain for two closely spaced pump pulse durations. By subtracting the corresponding Brillouin gain signals, the BGS can be reconstructed at a spatial resolution determined by the difference between the durations of the two pulses. Furthermore, the BGS keeps its narrow bandwidth dictated by the acoustic loss (~30 MHz), i.e., it gets rid of the inverse dependence of its bandwidth from the pulse duration as in conventional BOTDA sensors. Although the application of the differential pulse-pair method does not help when trying to reach the maximum acquisition speed (due to the requirement of two consecutive measurements for each pump/probe frequency shift), it is of great benefit when a submeter spatial resolution is required. In Ref. [68], a spatial resolution of 20 cm was achieved thanks to the use of a 52/50 ns pulse pair. Furthermore, the acquisition rate over a fiber length of 50 m was as high as 14 kHz, owing to the use of a frequency-agile probe wave and a limited (N = 51) number of frequency steps. We show in Figure 22 the Brillouin gain spectra acquired over an 80-cm vibrating fiber with only 5 averages. Despite the low number of averages, we can appreciate the very good SNR of these measurements, probably also thanks to the use of a PM fiber. 

## 7. Single-Shot BOTDA

**Figure 23:**
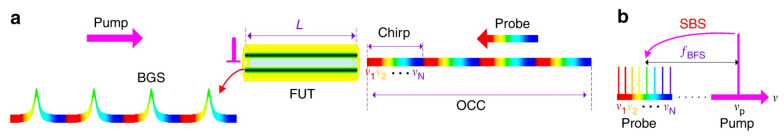
Principle of operation of the single-shot BOTDA based on the use of an OCC probe wave. (**a**) OCC probe wave is composed by several short optical chirp segments (**b**) Frequency relation between the pump pulse and the probe beam [70].

Ultimate performance in terms of probe frequency-sweep rate have been demonstrated in 2018 by Zhou et al. [70]. In their work, the authors have performed BGS measurements using a rapidly frequency-modulated probe wave, covering a large frequency range around the Stokes frequency with respect to the pump beam (see Figure 23). Compared to previous methods also relying on frequency-agile probe waves, the present method differs in that a fast and periodic modulation of the probe frequency is performed with a period of a few tens of nanoseconds, so that the entire BGS is real-time scanned in a time usually associated with a single resolution cell. As the probe frequency follows a sequence of optical chirps, the method has been named optical chirp chain (OCC) BOTDA.

**Figure 24:**
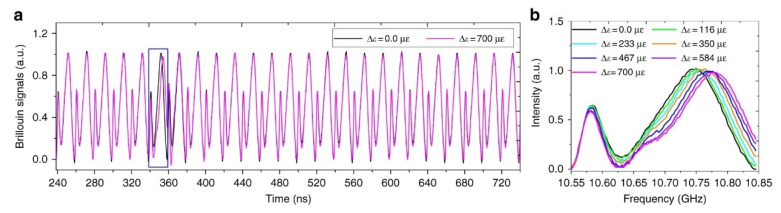
(**a**) Transmitted probe power as a function of time, showing a sequence of BGSs impressed on it due to interaction with the pump pulse. (**b**) Zoomed view of the portion of the probe signal corresponding to the segment of the fiber subjected to strain. The waveform has been acquired with an averaging factor of 200 and a pump pulse duration of 10 ns and an OCC duration of 20 ns [70].

Please note that the method is somewhat similar to the one presented in Ref. [69], with the fundamental difference that the linear sweep of the probe wave is compressed in the time domain, so that the pump pulse interacts with several frequency sweeps of the probe during its propagation along the fiber, and thus the BGS is acquired “on the fly”. The pump pulse duration must be shorter than the optical chirp segment, to not deteriorate the spatial resolution. The transmitted probe intensity, analyzed in the time domain, is shown in Figure 24 for a sawtooth modulation of the probe frequency. The plot shows that the probe intensity waveform presents a sequence of impressed BGSs, in a number equal to the number of OCCs injected into the FUT during the propagation of the pump over the entire fiber length. 

**Figure 25:**
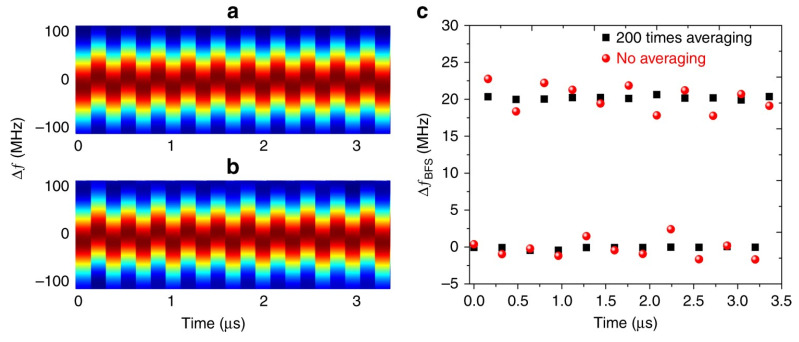
Time evolution of the BGS acquired over a 10-m long fiber for (**a**) averaging 200 times and (**b**) no averaging, where the BFS variation is simulated by periodically switching the probe frequency-sweep range. (**c**) Corresponding BFS evolution over time, with and without averaging [70].

Each impressed BGS is followed by a secondary (“ghost”) peak, due to the generation of an equivalent frequency near the BFS when the frequency is decreased from the highest frequency component to the lowest frequency component of the sawtooth waveform. The extraction of the BFS from each BGS relies on the time delay between each piece of the signal, and the corresponding piece of a reference signal previously acquired, which can be conveniently evaluated by cross-correlation techniques (see Figure 25b). Exploiting this method, dynamic strain measurements over a fiber length of 10 m, have been demonstrated at an exceptionally high sampling rate of 6.25 MHz. Figure 25 shows the time evolution of the BGS in a generic section of the fiber, obtained emulating a periodic, 20-MHz shift of the BGS though periodical shifting of the frequency sweep applied to the probe.

A successive optimization of the OCC method was proposed by the same authors in Ref. [71], which combines the frequency-agile probe with the differential pulse-pair method [29], and a pattern recognition algorithm. In such a case, the differential method was not added for spatial resolution enhancement, rather it was used to reduce the OCC modulation noise and avoid the self-phase modulation (SPM) effect. The OCC modulation noise arises from the uneven amplitude response for different frequency components, while SPM distorts the pump pulse waveform in long-range measurements. In Ref. [71], the temperature was measured over a fiber length of 100 km at a spatial resolution of 4 m. However, the measurement time was 5 s in this case, due to the long FUT and the relatively large averaging factor (*N_av_* = 2000).

The main drawbacks of the OCC method are the need for sophisticated, high sampling rate AWGs, and the underlying trade-off between spatial resolution, SNR, and dynamic range. In fact, the extension of the dynamic range requires the adoption of a longer chirp (for a fixed chirp rate), negatively influencing the spatial r6esolution. Similarly, the use of a steeper chirp leads to a time compression of the BGS impressed on the probe intensity, reducing the accuracy of the cross-correlation for a given sampling rate (unless more sophisticated extraction techniques such as those based on principal component analysis (PCA) are adopted, as in Ref. [71]). 

We finally observe that an acquisition rate close to the maximum rate dictated by the chosen fiber length (10 MHz), as reported in Ref. [70] for a 10-m fiber, means that no averaging has been performed. In other words, an adequate SNR was achieved even with single-shot acquisitions, thanks to a careful optimization of the setup (very narrow linewidth laser, PM FUT, careful choice of the injected optical powers, optical filtering of the amplified spontaneous emission (ASE) noise added by erbium-doped fiber amplifiers (EDFAs)). An optimized setup that does not require averaging, is very fast even when employed to conduct conventional BOTDA measurements. For example, for a conventional BOTDA scheme operated with no averaging, measurement over a 10-m fiber length and with 100 scanned frequencies gives an acquisition rate of 100 kHz, which is adequate in many applications.

## 8. Conclusions

The measurement of dynamic phenomena and the quantification of strain amplitude is a challenge for distributed optical fiber sensors. Rayleigh scattering-based configurations allow the detection of vibrations and their spectral analysis, but do not easily provide reliable quantitative measurements because of their limited linearity range and phase ambiguity [72]. On the other hand, Brillouin-based configurations exhibit an excellent linearity and can measure the absolute strain with an extended range. In this paper, we have reviewed some of the more interesting techniques proposed for dynamic sensing based on Brillouin scattering.

The more relevant aspects inherent performance of the presented methods are summarized in Table 1. Please note that the reported values are only indicative, and do not represent the ultimate performance of the various methods.

Apart from the specific technique applied, one major factor impacting the capability to perform high-speed measurements is the SNR: an optimized configuration with a higher SNR permits the reduction (or even elimination) of the need for averaging. Thus, it is fundamental to understand and optimize the principal factors influencing the SNR in Brillouin systems [73]. On the other hand, the relatively low strain sensitivity of the BFS (∼50 kHz/µε in conventional fibers), combined with the BGS intrinsic linewidth (∼30 MHz), makes improbable that Brillouin sensors will reach the nε sensitivity of Rayleigh-based sensors, even after that all the experimental parameters have been optimized. Therefore, possible future developments in the distributed dynamic sensing field in optical fibers may involve the simultaneous use of Rayleigh and Brillouin scattering into the same fiber [74], combining high sensitivity and adequate dynamic range. Another factor that should be taken into account, is that the present review only refers to configurations based on the SBS. Several configurations for dynamic sensing based on spontaneous Brillouin scattering have been proposed as well (e.g., [75,76]) but they have not been considered in this review, as spontaneous Brillouin scattering is intrinsically much weaker than SBS, which means that much more averages are required to satisfy the same SNR requirements. The use of SBS imposes the access to both ends of the fiber, which may be a limitation in some applications. Single-ended SBS configurations have been proposed as well, but generally are based on the use of some reflector at the end of the fiber [77,78], which means that these configurations are not fault tolerant, as a single breakage along the fiber prevents the measurement on the whole length. A recent demonstration of a truly single-ended BOTDA configuration [79] exploits the non-linear interaction between a Rayleigh-backscattered wave and a forward-traveling optical pump pulse, using sources separated by approximately one BFS. Such a configuration may be also adopted for dynamic sensing, for example by combining it with the SA method discussed in Section 4, to realize truly single-ended BOTDA dynamic strain measurements.

## Figures and Tables

**Table 1:** Summary of the main performance of fast Brillouin sensing methods.

	BOCDA [39]	SA-BOTDA [50,55]	Pump Power Independent SA-BOTDA [60,61]	Sweep-Free BOTDA [63]	Fast BOTDA [67,69]	Single-Shot BOTDA [70]
**Spatial resolution**	10′s of cm	~m	~m	10′s of m	~m	~m
**Sensing range**	10′s of m	100′s of m	100′s of m	100′s of m	100′s of m	10′s of m for high-speed measurements
**Acquisition rate**	~kHz	~kHz	~kHz	~kHz	10′s of Hz	~MHz
**Dynamic range**	100′s of MHz	∼30 MHz	∼30 MHz	100′s of MHz	100′s of MHz	100′s of MHz
**Main limitation(s)**	Only one (or a few) simultaneous sensing positions	Low dynamic range and pump power/ loss dependance	Low dynamic range	Low spatial resolution	Setup complexity	Trade-off between spatial resolution, SNR, and dynamic range
